# Genotoxic Effects of Tributyltin and Triphenyltin Isothiocyanates, Cognate RXR Ligands: Comparison in Human Breast Carcinoma MCF 7 and MDA-MB-231 Cells

**DOI:** 10.3390/ijms20051198

**Published:** 2019-03-09

**Authors:** Luba Hunakova, Eva Horvathova, Karolina Majerova, Pavel Bobal, Jan Otevrel, Julius Brtko

**Affiliations:** 1 Cancer Research Institute, BMC, Slovak Academy of Sciences, Dubravska cesta 9, 845 05 Bratislava, Slovak Republic; Eva.Horvathova@savba.sk (E.H.); Karolina.Majerova@savba.sk (K.M.); 2 Department of Chemical Drugs, Faculty of Pharmacy, University of Veterinary and Pharmaceutical Sciences Brno, Palackeho 1946/1, 612 42 Brno, Czech Republic; bobalp@vfu.cz (P.B.); otevrelj@vfu.cz (J.O.); 3 Institute of Experimental Endocrinology, BMC, Slovak Academy of Sciences, Dubravska cesta 9, 845 05 Bratislava, Slovak Republic; Julius.Brtko@savba.sk

**Keywords:** triorganotin isothiocyanates, breast cancer, cytotoxicity, apoptosis, DNA crosslinks

## Abstract

The cytotoxicity of two recently synthesized triorganotin isothiocyanate derivatives, nuclear retinoid X receptor ligands, was tested and compared in estrogen-receptor-positive MCF 7 and -negative MDA-MB-231 human breast carcinoma cell lines. A 48 h MTT assay indicated that tributyltin isothiocyanate (TBT-ITC) is more cytotoxic than triphenyltin isothiocyanate (TPT-ITC) in MCF 7 cells, and the same trend was observed in the MDA-MB-231 cell line. A comet assay revealed the presence of both crosslinks and increasing DNA damage levels after the 17 h treatment with both derivatives. Differences in cytotoxicity of TBT-ITC and TPT-ITC detected by FDA staining correspond to the MTT data, communicating more pronounced effects in MCF 7 than in the MDA-MB-231 cell line. Both derivatives were found to cause apoptosis, as shown by the mitochondrial membrane potential (MMP) depolarization and caspase-3/7 activation. The onset of caspase activation correlated with MMP dissipation and the total cytotoxicity more than with the amount of active caspases. In conclusion, our data suggest that the DNA damage induced by TBT-ITC and TPT-ITC treatment could underlie their cytotoxicity in the cell lines studied.

## 1. Introduction

A class of triorganotin compounds act as nuclear retinoid X receptors (RXR) agonists, due to their capability to bind to the ligand-binding domain of RXR subtypes [1,2,3], and thus, they function as potent transcriptional activators [4,5]. In general, triorganotins actions are occurring predominantly through target gene(s)-mediated pathways. They are rather stable in a water solution [6] and cause a number of molecular interactions, also with the reproductive system in mammals [7]; as potent environmental obesogens, they promote adipocyte differentiation [2,8], and as environmental pollutants, they can induce DNA damage [9,10]. On the other hand, organometallic compounds and metal complexes have been gaining growing importance in oncology [11], and among organotin compounds, triorganotin derivatives in particular have demonstrated cytotoxic properties against a number of tumor cell lines [5,12,13]. The cytotoxicity of tributyltin (TBT) in neuroblastoma cells [14] has been described at very low concentrations (0.1–1 μM), while in mammalian endothelial cells the IC50 value of TBT exceeded 1 μM concentration [15]. It has been suggested that some of the organotin compounds might be involved in cancer treatment via different mechanisms at the molecular level [16,17], thus opening up a new research subarea on organotin compounds [5,18,19].

Numerous mechanistic studies have demonstrated the anticancer effects of isothiocyanates (ITCs). In various cancer types, they were found to suppress tumor growth by generating reactive oxygen species or inducing cell cycle arrest, leading to apoptosis [20]. Recent research has been aimed at the possible therapeutic benefits of natural [21] or synthetic ITCs [22,23], in conjunction with standard anticancer drugs. Sulforaphane’s anticancer efficacy has been shown to be associated with the reversal of several biological characteristics connected with the epithelial‒mesenchymal transition (EMT) or implicated in the matrix degradation and extracellular proteolysis, as well as with reduced production of pro-inflammatory cytokines and pro-angiogenic growth factors in the MDA-MB-231 cell line [24]. Thus, ITCs of either natural or synthetic origin may be considered to be promising anticancer agents.

**Figure 1:**
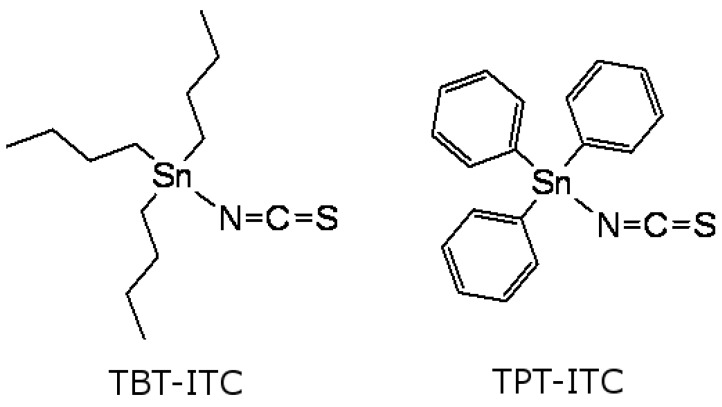
Structures of tributyltin isothiocyanate (TBT-ITC) and triphenyltin isothiocyanate (TPT-ITC).

The goal of our study was to combine the anticancer properties of two different chemical structures previously described in the literature, isothiocyanates and triorganotins, merged into the following molecules: (i) tributyltin isothiocyanate and (ii) triphenyltin isothiocyanate (Figure 1), as organometallic compounds have been gaining growing importance in experimental oncology [11,25]. Both of the abovementioned compounds are commercially inaccessible. Since no literature data have been available concerning the genotoxicity of triorganotin isothiocyanates up until now, TBT-ITC and TPT-ITC were used to test the hypothesis that these compounds may exert genotoxic effects in human breast carcinoma MCF 7 and MDA-MB-231 cells. To test this hypothesis, we used the comet assay, detecting DNA damage to study, distinguish, and compare frank DNA breaks, alkali-labile sites, and crosslinks with those caused by the well-known chemotherapeutic drug cisplatin. MTT tests detecting cytotoxicity, flow cytometry, and live cell imaging were used to assess the type of cell death. Their non-genotoxic effects at lower concentrations will be reported in a subsequent paper.

## 2. Results

**Figure 2:**
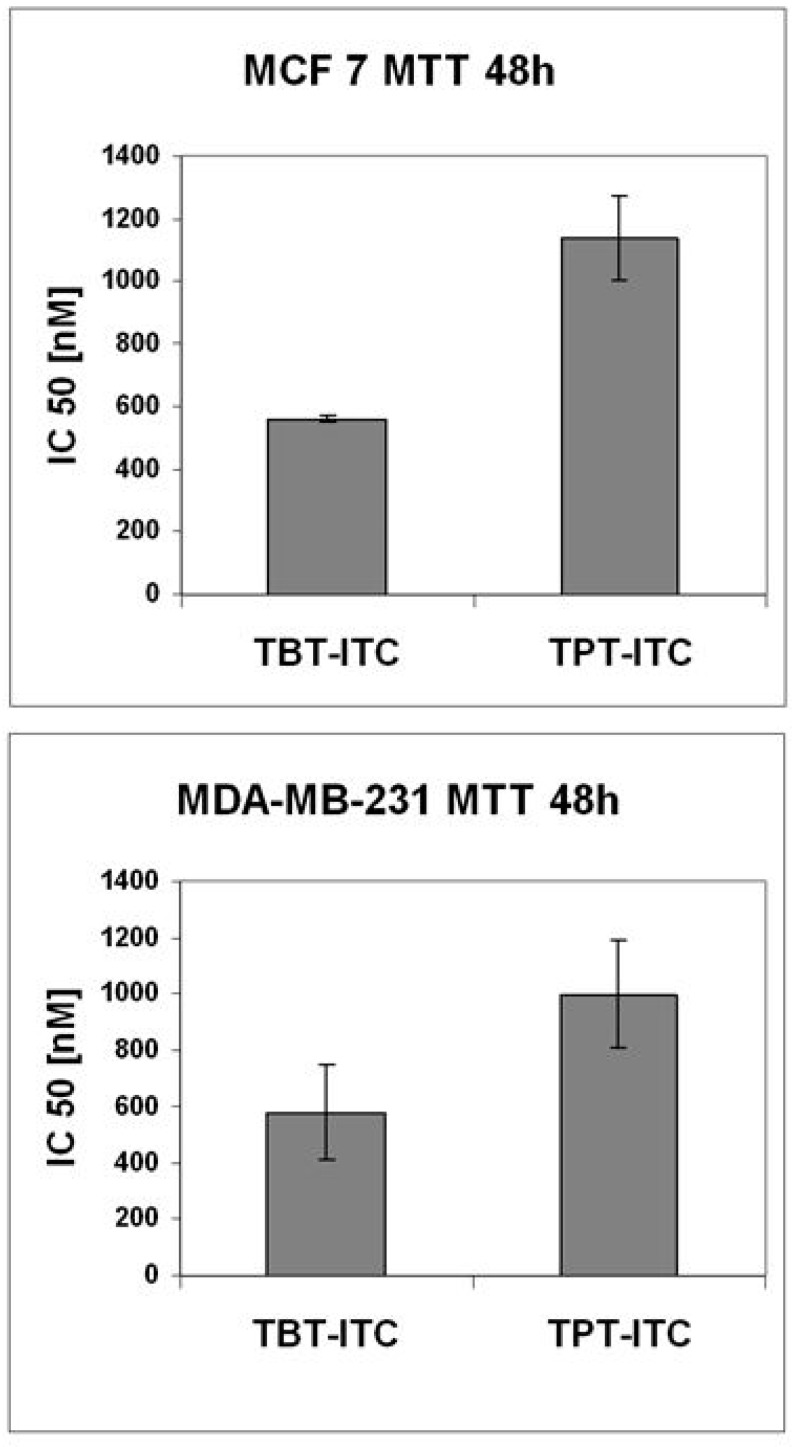
Cytotoxicity of tributyltin and triphenyltin isothiocyanates in MCF 7 and MDA-MB-231 cells. The concentrations of drugs that inhibited cell survival to 50% (IC50) at 48 h measured by MTT assay and determined by Calcusyn software are expressed as the means ± standard deviation (SD) of three to five independent experiments.

The tested compounds showed cytotoxicity against both human breast carcinoma cell lines, with TBT-ITC tending to be more cytotoxic than TPT-ITC. Although differences in 48 h IC50 values in the MDA-MB-231 cell line did not reach statistical significance, in MCF 7 cells the 48 h IC50s differed significantly and almost doubled for TPT-ITC in comparison with TBT-ITC (Figure 2).

**Figure 3:**
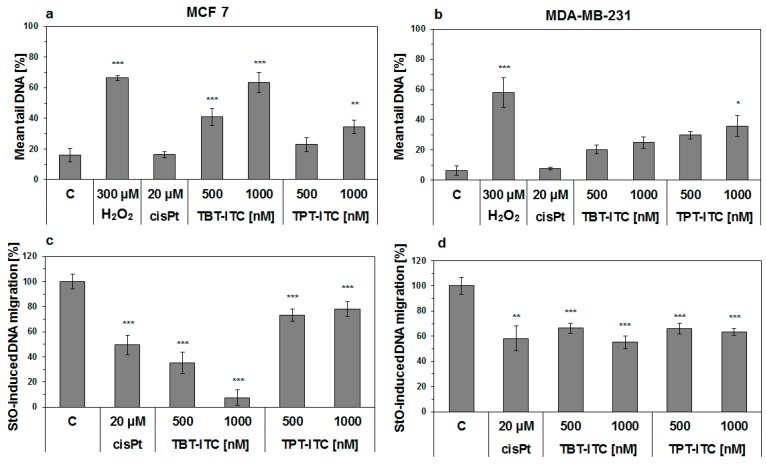
DNA damage (alkaline comet assay) caused by treatment with 500 and 1000 nM TBT-ITC and TPT-ITC in human breast cancer MCF 7 (**left**) and MDA-MB-231 cells (**right**) expressed either as a mean percentage of tail DNA (DNA-damaging 300 μM hydrogen peroxide, H_2_O_2_, was used as a positive control) (**a**,**b**); or as a percentage of StO-induced DNA migration (crosslinking 20 μM cisplatin, cisPt, was used as a positive control) (**c**,**d**). Results are presented as the means of three independent experiments ± standard deviation (SD). Statistically significant differences * *p* < 0.05, ** *p* < 0.01, *** *p* < 0.001 in comparison to the negative (untreated) control (C).

With the conventional comet assay, we detected dose-dependent increases of DNA damage levels after the treatment with ITC derivatives in both human breast carcinoma cell lines, which were more pronounced and statistically significant in MCF 7 cells. This tendency of increasing frank DNA breaks and alkali-labile sites was also observed in MDA-MB-231 cells with statistical significance at the highest 1000 nM concentration of TPT-ITC (Figure 3a,b). A comet assay also revealed the presence of crosslinks in the population of attached cells after the 17 h treatment with both ITC derivatives indicating dose-dependent activity of TBT-ITC in both human breast carcinoma cells, in contrast to TPT-ITC, where we did not observe increased dose-dependent crosslinks and their levels were lower in comparison to TBT-ITC and cisPt in the MCF 7 cell line (Figure 3c,d).

**Figure 4:**
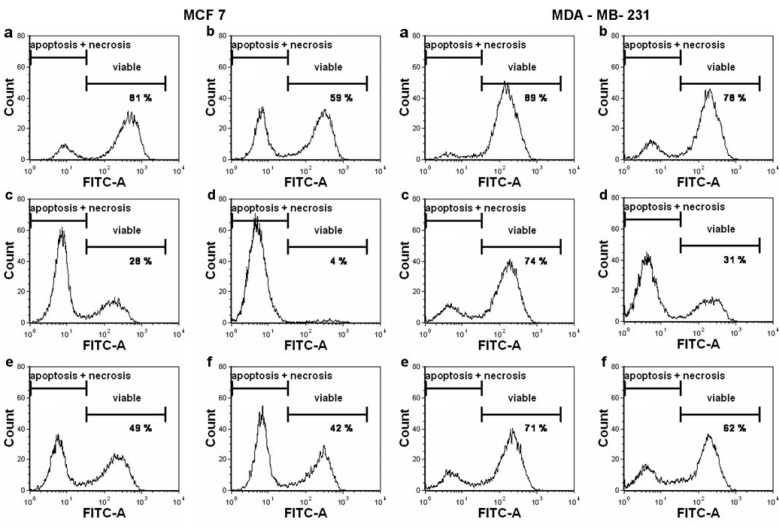
Apoptosis and necrosis induction by triorganotin isothiocyanate derivatives in MCF 7 and MDA-MB-231 cells measured by flow cytometry (FDA/PI staining). The proportion of viable (FDA+/PI-), apoptotic (FDA-/PI-), and necrotic (FDA-/PI+) cells is illustrated in histograms after following treatment: (**a**) control, (**b**) Taxol 1 µM (positive control), (**c**) TBT-ITC 500 nM, (**d**) TBT-ITC 1 µM, (**e**) TPT-ITC 500 nM, and (**f**) TPT-ITC 1 µM. The data presented are representative histograms of three independent experiments.

The cytotoxicity determined by FDA staining showed a reduction of viable cell population in both cell lines in a dose-dependent manner, which was accompanied by an increase in apoptotic and necrotic populations (Figure 4). For TPT-ITC, the increase in apoptotic and necrotic cell populations with a concomitant decrease of viable cells was not as pronounced as for TBT-ITC, but still detectable. Differences in the cytotoxicity of TBT-ITC and TPT-ITC detected by FDA staining seem to correspond with the MTT results, showing more pronounced effects in MCF 7 than in MDA-MB-231 cell line.

**Figure 5:**
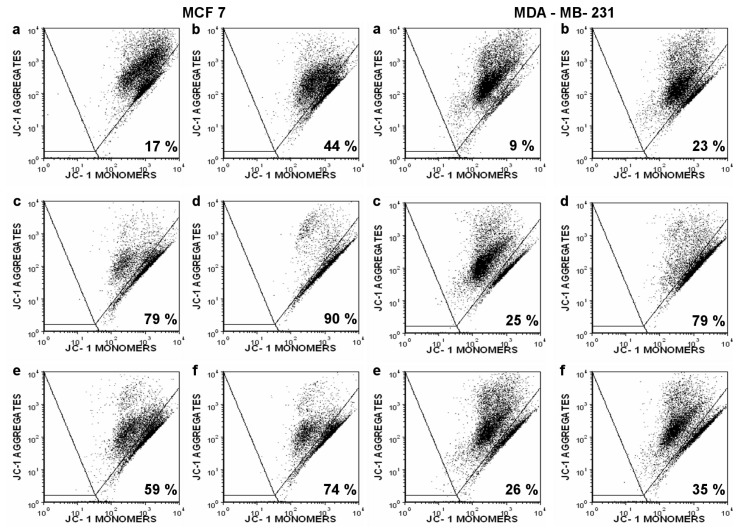
The mitochondrial membrane potential disruption by triorganotin isothiocyanate derivatives in MCF 7 and MDA-MB-231 cells measured by flow cytometry (JC-1 staining). The percentage of cells with depolarized Δψm (JC-1 monomers) is indicated in the right lower quadrant after following treatment: (**a**) control, (**b**) Taxol 1 µM (positive control), (**c**) TBT-ITC 500 nM, (**d**) TBT-ITC 1 µM, (**e**) TPT-ITC 500 nM, and (**f**) TPT-ITC 1 µM. The data presented are representative dot plots of three independent experiments.

**Figure 6:**
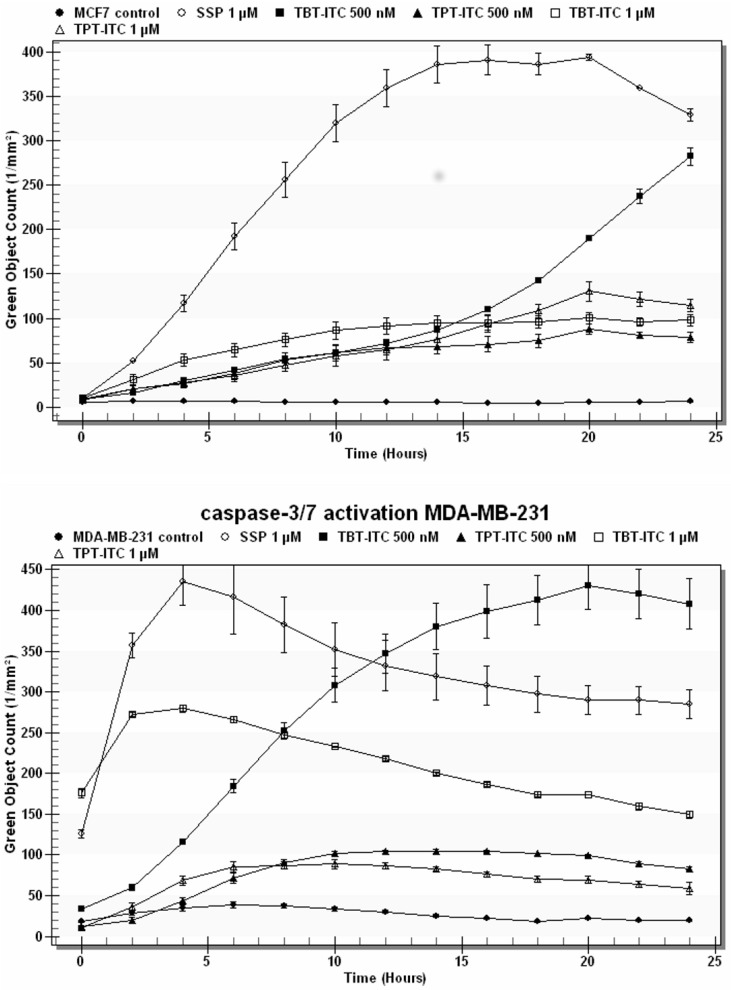
Caspase-3/7 activation in human breast cancer cells. Caspase-3/7-positive objects stained by CellPlayer™ Kinetic Caspase-3/7 Apoptosis Assay Reagent were measured over 24 h in response to increasing concentrations of TBT-ITC and TPT-ITC derivatives. SSP (1 μM) was used as a positive control.

Both derivatives caused apoptosis, as shown by the drop of mitochondrial membrane potential (MMP) (Figure 5) and caspase-3/7 activation (Figure 6). Mitochondrial membrane depolarization was stronger and the differences between TBT-ITC and TPT-ITC were more prominent in the MCF 7 cell line than in MDA-MB-231. The decrease of MMP was comparable to the 500 nM concentration of both compounds in MDA-MB-231 cells. Onset of caspase-3/7 activation was sooner in MDA-MB-231 than in MCF 7 cells and a 1 µM concentration of both compounds activated executive caspases more quickly (within 4‒5 h) than the 500 nM concentration (10‒15 h) in this cell line. In MCF 7 cells, both concentrations of TBT-ITC showed similar dynamics of caspase activation to the MDA-MB-231 cell line; however, the 500 nM and 1 µM concentrations of TPT-ITC did not differ dramatically.

## 3. Discussion

Triorganotin compounds have been gaining importance in oncology due to their cytotoxic properties against various human cell lines including breast carcinoma [11,13,16,25]. Recently, we studied selected Sn- and Ge-triorganometallic compounds and have reported the different cytotoxicity and modulation of migration in triple-negative breast cancer cell line MDA-MB-231 [17]. Also, the in vitro effects of selected triorganotin ligands of nuclear retinoid X receptors have been studied in human MCF 7 breast cancer cells [19].

In this study, known anticancer/genotoxic properties of two different “molecule parts,” (i) triorganotin and (ii) isothiocyanate, combined into recently synthesized (commercially inaccessible) tributyltin isothiocyanate (TBT-ITC) and triphenyltin isothiocyanate (TPT-ITC), underwent investigation of their cytotoxic effects in both human estrogen-receptor-positive MCF 7 and human triple-negative MDA-MB-231 breast carcinoma cell lines.

The cytotoxicity of both compounds has been recently reported in L1210 mice leukemia cells at a submicromolar concentration independently of P-glycoprotein overexpression [26]; this is consistent with our present findings in human carcinoma cells. Additionally, triorganotins have shown higher cytotoxicity in leukemia S cells when compared to normal murine pre-B cells PB-1, which demonstrates their selective action on neoplastic cells [26].

In MCF 7 cells, TBT-ITC was more cytotoxic than TPT-ITC after 48 h treatment, while in the MDA-MB-231 cell line, the same tendency did not reach statistical significance in the MTT test. This could be caused by a slightly higher potency of TPT-ITC in MDA-MB-231 than in MCF 7 cells (with a *p* value of 0.291 using the Fisherian approach). Our findings could be perceived as a consequence of the genotoxicity detected by the comet assay after 17 h treatment. Lorenzo et al. [27] stated that the comet assay may detect the earliest stages of apoptosis as “hedgehog“ comets with almost all DNA in the tail (>80%). However, in our conventional comet analyses performed on the population of attached (non-apoptotic) cells, treatment of MCF 7 cells with 500 or 1000 nM TBT-ITC induced 40.9% or 63.4% of tail DNA, respectively, and heavily damaged comets were not present. The levels of crosslinks induced by TBT-ITC were significantly higher than those induced by TPT-ITC in MCF 7, but not in MDA-MB-231 cells. Similarly, cell death analysis using FDA staining showed a reduction in the viable cell population in both cell lines in a dose-dependent manner, while for TPT-ITC the decrease in viable cells was not as pronounced as for TBT-ITC, although still detectable. Differences in the cytotoxicity of TBT-ITC and TPT-ITC detected by FDA staining seem to correspond to the MTT results, with more pronounced effects for MCF 7 in comparison to the MDA-MB-231 cell line. In our previous studies with organotin compounds, we found similar phenomena with tributyltin chloride (TBT-Cl) and triphenyltin chloride (TPT-Cl) [13]. As we discussed there, the observed differences in cytotoxicity have been attributed to different modulation of the RXRalpha nuclear receptor in the triple-negative MDA-MB-231 cell line. Based on this, we hypothesize that differences in sensitivity of both cell lines to the new (ITC) compounds could be due to different modulation of the main components of the retinoid/estrogen signaling cross-talk. When we take into consideration the relationship between increased levels of DNA damage (observed in attached cells) and the decreased cell viability induced by TBT-ITC and TPT-ITC in the MCF 7 cell line (and the same trend, although not significant, in MDA-MB-231 cells), it could be hypothesized that the DNA damage by TBT-ITC and TPT-ITC treatment contributes to their cytotoxicity in the cell lines studied. Similar effects of cis- and carboplatin on primary or recurrent epithelial ovarian cancer biopsies were observed by Unger et al. [28], unlike gemcitabine or adriamycin, in which DNA-damaging activities have shown no correlation with cytotoxicity, or the correlation was low. For both of these cytostatics, multiple molecular effects are known that could influence cytotoxicity, e.g., radical formation, inhibition of topoisomerase II or helicases, and modulation of DNA/RNA synthesis and DNA repair [29,30]. 

Although MMP dissipation was stronger and the differences between TBT-ITC and TPT-ITC were more prominent in the MCF 7 cell line than in MDA-MB-231, this trend was not observed in caspase-3/7 activation. First of all, the onset of caspase activation occurs later in the MCF 7 cell line in comparison with MDA-MB-231 cells, even for positive control SSP. Also, the total amount of activated caspases is lower for all used compounds in MCF 7 cells. This may be a consequence of the fact that MCF 7 cells lack caspase-3 and caspase-7 may make up for deficits in caspase-3 function for mitochondrial-dependent apoptosis in these cells [31].

In MDA-MB-231 cells, we found a comparable decrease of MMP for the 500 nM concentration of both compounds; this is related to the levels of crosslinks, but not to the amount of activated caspases-3/7. On the other hand, the dynamics of caspase activation was comparable for a 500 nM concentration of the tested compounds. Similarly, a 1 μM concentration of TBT-ITC activated executioner caspases more quickly than the same concentration of TPT-ITC, which corresponded well to the higher MMP dissipation induced by 1 µM TBT-ITC in this cell line. Thus, it seems that the onset of caspase activation correlates with MMP depolarization and the total cytotoxicity more than the amount of active caspases. This finding is supported by our previous data [13] that showed that the onset of TPT-Cl-induced caspase-3/7 activation was delayed in comparison with TBT-Cl corresponding to the cytotoxicity. Since many organic compounds from the ITC group are known to decrease cell viability and proliferation, induce G2/M cell cycle arrest, and promote apoptosis through the activation of caspases 3, 8, and 9 [32], we suppose that two different “molecule parts” (triorganotin and isothiocyanate groups) merged into “one molecule” may thus yield a combined cytotoxic effect.

In general, triorganotin molecules offer promissing possibility for subsequent derivatization. Moreover, various donor ligands directly linked to the tin atom may also result in the development of novel triorganotin compounds with even higher antiproliferative activity against a variety of solid and hematologic cancer cells [11]. Our recent data have shown slightly higher cytotoxicity in hematological than in solid cancer cells [26].

To summarize our results, we demonstrated that the DNA damage caused by two triorganotin isothiocyanates (TBT-ITC and TPT-ITC) contributes to their cytotoxicity in human estrogen-receptor-positive and triple-negative breast cancer cell lines.

## 4. Materials and Methods

### 4.1. Reagents

Fluorescein diacetate (FDA), propidium iodide (PI), 3-[4,5-dimethylthiazol-2-yl]-2,5-diphenyl tetrazolium bromide (MTT), Taxol (TX), styrene oxide (StO), and ethidium bromide (EtBr) were obtained from Sigma Chemical Co. (Schnelldorf, Germany). Cisplatin (cisPt) was acquired from Lachema (Czech Republic). Bovine serum albumin (BSA) was purchased from AppliChem GmbH (Denmark), Staurosporin (SSP) from Santa Cruz Biotechnology Inc. (Santa Cruz, CA, USA), JC-1 (5,5′,6,6′-tetrachloro-1,1′,3,3′-tetra-ethylbenzimidazolylcarbocyanine iodide) from Invitrogen (Thermo Fisher Scientific, USA). CellPlayer™ Kinetic Caspase-3/7 Apoptosis Assay Reagent was purchased from Essen BioScience, UK. Tributyltin isothiocyanate (TBT-ITC) and triphenyltin isothiocyanate (TPT-ITC) were prepared by refluxing alcohol solutions of the respective tin chlorides with KSCN used in excess (1.5 equiv.), as previously described [33,34]. The obtained crude products were purified either by fractional bulb-to-bulb distillation (TBT-ITC: bp 160 °C, 0.25 mm [33]) or recrystallization from CH_2_Cl_2_-n-hexane (TPT-ITC: mp 165-167 °C [34]). The structures of the prepared compounds were confirmed by IR and NMR techniques. IR spectra were recorded on a SmartMIRacle ATR Zn/Se for Nicolet Impact 410 FT-IR (Thermo Scientific, Langenselbold, Germany). NMR spectra were measured on a JEOL ECZR-400 MHz spectrometer (Jeol Ltd., Akishima, Tokyo, Japan). Experiments were carried out at 25 °C; chemical shifts are reported in δ parts per million (ppm) and J values in Hz. The residual solvent signals of CDCl_3_ were used as a reference.

TBT-ITC and TPT-ITC spectral characteristics are shown in our recently published work [26]. The purities of the prepared triorganotin compounds (tributyltin isothiocyanate and triphenyltin isothiocyanate) were better than 97%, as determined by NMR.

### 4.2. Cells and Treatment

The MCF 7 and MDA-MB-231 human breast cancer cell lines were routinely cultured in an RPMI 1640 medium supplemented with 10% heat-inactivated FCS, 2 mM l-glutamine, 100 μg/mL penicillin, and 50 μg/mL streptomycin; 0.5 × 10^6^ cells/mL were cultured in 96-, 24- or six-well plates (Greiner, Germany). The cultures were passaged twice a week after reaching a cell density of 0.8‒1.0 × 10^6^ cells/ml. Cells were plated at 3‒6 × 10^4^ cells/cm^2^ density on the day before treatment and exposed to various concentrations of TBT-ITC and TPT-ITC for the time indicated. Stock solutions of tested compounds were originally dissolved in ethanol, and an equal volume of ethanol (final concentration < 0.02%) was added to the control cells.

### 4.3. Cytotoxicity Assay

The effect of tested triorganotin-ITC derivatives on survival of breast cancer cells was determined by MTT assay [35]. Cells were seeded at a 1‒2 × 10^3^ cell density in 96-well culture plates. Each dose of tested compounds (added in the volume of 50 μL) was tested in triplicate or quadruplicate. After 48 h, the cells were incubated with 50 μL of MTT (1 mg/mL) and left in the dark at 37 °C for an additional 4 h. Thereafter, medium was removed, the formazan crystals were dissolved in 200 μL of DMSO, and the absorbance was measured at 540 and 690 nm with an xMark™ Microplate Spectrophotometer (Bio-Rad Laboratories, Inc.). The concentration of drug that inhibited cell survival to 50% (IC50) was determined by Calcusyn software (version 1.1, Biosoft).

### 4.4. Alkaline Single-Cell Gel Electrophoresis (Alkaline Comet Assay)

Assessment of DNA damage in breast cancer cells was performed by the alkaline comet assay as described by Slamenova et al. [36]. MCF 7 and MDA-MB-231 cells were treated with the compounds tested (TBT-ITC and TPT-ITC both at concentrations of 500 and 1000 nM) in a supplemented RPMI 1640 medium in six-well plates for 17 h at 37 °C. Then the medium was removed and the cells were washed with PBS, trypsinized, embedded into gels, and processed for the comet assay. Dead and detached cells were not included in the DNA damage assessment. In the conventional comet assay, the positive control was treated with 300 μM hydrogen peroxide (H_2_O_2_) in PBS at 4 °C for 5 min in the dark. In the modified comet assay for detection of crosslinks, the incubation of the cells with styrene oxide (StO) was performed [21] and cisplatin (20 μM cisPt for 17 h treatment) was used as a positive control. Conventional as well as modified comet assays were carried out to study, distinguish, and compare frank DNA breaks with alkali-labile sites and crosslinks. We examined 35‒60 EtBr-stained nucleoids per microgel/slide (prepared in triplicate per sample) in one electrophoresis run with a Carl Zeiss AxioImager.Z2 fluorescence microscope using a computerized image analysis Metafer 5 (MetaSystems GmbH, Germany). The percentage of DNA in the tail was used as a parameter for the measurement of DNA damage. In experiments for the detection of crosslinks, the values for DNA damage are given as percentages of StO-induced DNA migration calculated according to the modified formula suggested by Hunakova et al. [21]. Results are presented as the means of three independent experiments ± standard deviation (SD). The data were analyzed using SPSS 23.0 software. The Shapiro‒Wilk test was used to test the normality of distribution. Normally distributed data differences between the two groups were tested by Student’s *t*-test or Mann‒Whitney *U* test, if equal variances were not assumed. Differences between more than two groups were assessed by one-way analysis of variance (ANOVA) followed by the Bonferroni or Tamhane’s test for multiple comparisons. Non-normally distributed data were tested by Kruskal‒Wallis *H* test followed by multiple comparisons. Differences of *p* < 0.05 were considered statistically significant.

### 4.5. Flow Cytometry

Exponentially growing cells at 70‒80% confluence were treated at time 0 with the tested compounds for 17 h. Apoptotic cell enumeration was done according to the number of cells with the cell membrane impermeable to PI (PE-A) and low FDA fluorescence (FITC-A); necrotic cells were determined as the cell membrane permeable for PI (PE-A). As for FDA/PI staining [37], both cell lines were collected as described above. Pooled cells were washed twice with cold PBS. Approximately 5 × 10^5^ cells were resuspended in 400 μL of PBS/0.2% BSA containing 10 nM of FDA (from a 5 mM stock in DMSO) for 30 min at room temperature. Then cells were cooled and 4 μL of PI (1 mg/mL) was added. Finally, cells were measured using Canto II (Becton Dickinson) flow cytometer and analyzed by FCS Express 4.0 (De Novo Software).

To identify the type of cell death, apoptosis/drop of MMP was also measured by JC-1 staining because of JC-1’s specificity regarding the mitochondrial membrane. Briefly, control and treated cells were harvested, washed twice with PBS, and incubated with 400 μL of PBS/0.2% BSA containing 4 mM of JC-1 for 30 min (37 °C; 5% CO_2_ atmosphere). For each sample, 10,000 cells were examined on an FL-1 (530 nm, JC-1 monomers) versus FL-2 (585 nm, JC-1 aggregates) dot plot using a Canto II cytometer. JC-1 has dual emission depending on the state of the Δψm, forming aggregates with a high fluorescence of 585 nm in cells (which indicates a normal Δψm). Loss of mitochondrial membrane integrity results in a reduction in 585 nm fluorescence with a concurrent gain in 530 nm fluorescence as the dye shifts from an aggregate to a monomeric state [38]. The percentage of cells with depolarized MMP was determined by using FCS Express 4.0 (De Novo Software).

### 4.6. Caspase-3/7 Assay

Caspase-3/7 activation was assessed by CellPlayer™ Kinetic Caspase-3/7 Apoptosis Assay Reagent (Essen BioScience), which couples the activated caspase-3/7 recognition motif (DEVD) with NucView™488, a DNA intercalating dye. When added to a tissue culture medium, this inert, non-fluorescent substrate crosses the cell membrane and is cleaved by activated caspase-3/7, resulting in the release of the DNA dye and green fluorescent staining of nuclear DNA. Cells were treated with SSP (1 µM), TBT-ITC, and TPT-ITC (500 and 1000 nM) in triplicate in a 96-well plate. Kinetic activation of caspase-3/7 was monitored morphologically using live cell imaging and quantified using the IncuCyte™ FLR object counting algorithm (Essen BioScience).

## 5. Conclusions

Two different “molecule parts” (triorganotin and isothiocyanate) were successfully merged into “one molecule,” generating a combined cytotoxic effect. In our work we showed that the DNA damage induced by these compounds (TBT-ITC and TPT-ITC) could contribute to their cytotoxicity in human estrogen-receptor-positive and triple-negative breast cancer cell lines.

## Abbreviations

BSA	Bovine serum albumin
cisPt	Cisplatin
DMSO	Dimethylsulfoxide
EMT	Epithelial‒mesenchymal transition
ER	Estrogen receptor
EtBr	Ethidium bromide
FCS	Fetal calf serum
FDA	Fluorescein diacetate
FITC	Fluorescein isothiocyanate
ITC	Isothiocyanate
MCF 7	Estrogen-receptor-positive human breast cancer cell line
MDA-MB-231	Estrogen-receptor-negative human breast cancer cell line
MMP, Δψm	Mitochondrial membrane potential
MTT	3-[4,5-dimethylthiazol-2-yl]-2,5-diphenyltetrazolium bromide
PBS	Phosphate-buffered saline
PI	Propidium iodide
RA	All-*trans* retinoic acid
RAR	Nuclear all-*trans* retinoic acid receptor
RXR	Nuclear retinoid X receptor
SSP	Staurosporin
StO	Styrene oxide
TBT-Cl	Tributyltin chloride
TBT-ITC	Tributyltin isothiocyanate
TPT-Cl	Triphenyltin chloride
TPT-ITC	Triphenyltin isothiocyanate
TX	Taxol
